# Thoracoscopic double-flap reconstruction for esophagogastric junction cancer: A case report

**DOI:** 10.1016/j.ijscr.2020.01.026

**Published:** 2020-01-27

**Authors:** Manato Ohsawa, Yoichi Hamai, Manabu Emi, Kazuaki Tanabe, Morihito Okada

**Affiliations:** a Department of Surgical Oncology, Hiroshima University, 1-2-3-Kasumi, Minami-ku, Hiroshima City, Hiroshima 734-8551, Japan; b Department of Gastroenterological and Transplant Surgery, Hiroshima University, 1-2-3-Kasumi, Minami-ku, Hiroshima City, Hiroshima 734-8551, Japan

**Keywords:** EGJ, esophagogastric junction, ESD, endoscopic submucosal dissection, Oesophagogastric junction cancer, Siewert adenocarcinoma, Oesophagogastrostomy, Thoracoscopic, Double-flap reconstruction

## Abstract

**Background:**

An anti-reflux anastomosis “double-flap technique” was recently used to resolve severe reflux esophagitis after intrathoracic esophagogastrostomy performed following proximal gastrectomy and lower esophagectomy, for esophagogastric junction (EGJ) cancer. We describe thoracoscopic reconstruction procedure performed by using the “double-flap” technique, which involves the creation of seromuscular flap under direct vision. This case report aimed to report the usefulness of this intrathoracic anastomosis procedure, as it may be difficult to perform double-flap technique with intraperitoneal manipulation in EGJ cancer cases.

**Presentation of case:**

A 58-year-old man was diagnosed with Siewert type II EGJ cancer. We performed laparoscopic proximal gastrectomy, lower esophagectomy, and thoracoscopic esophagogastrostomy using the anti-reflux double-flap technique in the prone position. This was achieved after careful dissection in the plane between the muscular and submucosal layers prior to replacing the remnant stomach into the abdominal cavity. The postoperative course was uneventful, with no symptoms of esophageal reflux after 21 months of surgery, even without medications.

**Discussion:**

This procedure offers the advantage of minimal invasiveness and ensures adequate surgical margins when lower esophageal incisions are required. This minimally invasive procedure achieves anastomosis using the complete hand-sewn method to prevent reflux, under a good surgical field of view for dissection of the lower esophagus and mediastinal lymph nodes.

**Conclusions:**

This procedure is very useful due to its minimal invasiveness, ease of thoracic procedure, and prevention of reflux in patients with EGJ cancer. To our knowledge, this is the first report of thoracoscopic esophagogastrostomy performed using the double-flap technique for EGJ cancer.

## 1 Introduction

The present work has been reported in line with the SCARE criteria [1]. Although intrathoracic esophagogastrostomy is needed following proximal gastrectomy and lower esophagectomy for patients with esophagogastric junction (EGJ) cancer, it is frequently accompanied by severe reflux esophagitis. An anti-reflux anastomosis “double-flap technique” was recently performed after proximal gastrectomy for early upper gastric cancer [[2], [3], [4]]. We describe the use of this anastomotic technique with a complete thoracoscopic approach and the patient in the prone position as a reconstruction procedure. This case report aimed to report the usefulness of this intrathoracic anastomosis procedure, as it may be difficult to perform double-flap technique with intraperitoneal manipulation in EGJ cancer cases.

## 2 Presentation of case

**Fig. 1:**
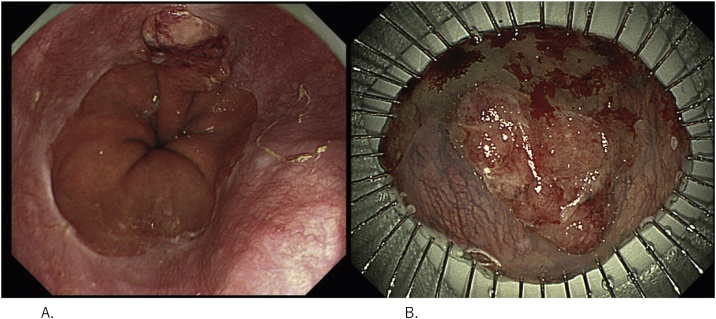
Endoscopic examination and endoscopic submucosal dissection (ESD) specimen. **(A)** Esophagogastric junction cancer originating from the short segment of the Barrett’s esophagus. **(B)** ESD specimen.

A 58-year-old man was referred to our department from his primary care hospital for treatment of EGJ cancer. He was asymptomatic. His medical and family history was unremarkable. On physical examination, his abdomen was soft and flat, with no tenderness. Biochemistry results and tumor marker levels were within normal limits. Endoscopic examination showed a flat, elevated tumor measuring approximately 25 mm in diameter located at the EGJ and abdominal esophagus, originating from the short segment of Barrett’s esophagus (Fig. 1A). Histological examination of the biopsied specimens led to the diagnosis of a well-differentiated adenocarcinoma. He was diagnosed with Siewert type II Barrett’s esophagus cancer without lymph node or distant metastases. He underwent endoscopic submucosal dissection (ESD) (Fig. 1B). Pathologically, the tumor had invaded the submucosal layer by 120 μm and was accompanied by lymphatic invasion. Additional treatment was indicated, and he was referred to our surgical department. Subsequently, we performed proximal gastrectomy and lower esophagectomy. The treatment strategies followed in this case were discussed and decided upon by a multidisciplinary tumor board at our institute.

First, a proximal gastrectomy and lower esophagectomy were performed with the patient in the supine position and using five trocars with the laparoscopic approach. We processed the greater and lesser curvatures of the stomach and performed resection at the upper part of the stomach. Intraoperative upper endoscopy was performed, and the esophagus was cut immediately above the scar after ESD at approximately 3 cm from the EGJ. However, we could not tow the esophagus sufficiently to perform anastomosis inside the abdominal cavity and judged that laparoscopic esophagogastrostomy was impossible. Because this was preoperatively predicted to a certain extent, we decided to perform intrathoracic anastomosis using the double-flap technique via the thoracoscopic approach.

The resected specimen and remnant stomach were removed from the abdomen via a small, expanded wound at the navel port. A lateral H-type incision (height 3.5 cm × width 2.5 cm) was made 3–4 cm below the tip of the remnant stomach, and a “double door” seromuscular flap was created under direct vision [3]. After carefully peeling the muscle layer from the submucosal layer, a “double-flap” was made, and the remnant stomach was returned to the abdomen. After suture fixation of the esophageal stump and remnant stomach, the patient was moved to the prone position.

**Fig. 2:**
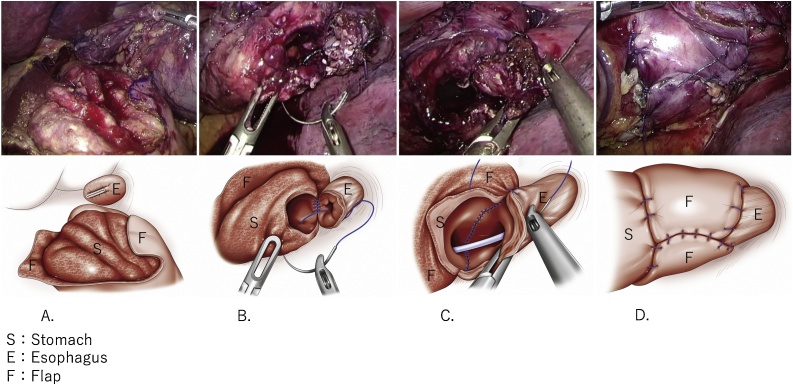
Double-flap technique. **(A)** Seromuscular double flaps: The muscle layer is peeled from the submucosal layer. **(B)** Posterior wall anastomosis: Esophageal walls are sutured to the gastric walls, which is peeled from the muscle layer. **(C)** Anterior wall anastomosis. **(D)** Closure and flap securement with sutures: Anastomosis is covered with the double flap.

Thoracoscopic reconstruction was performed using five trocars. Esophagectomy was performed after confirming that there was no problem with the upper gastrointestinal endoscope during the operation. During removal of the lower esophagus via thoracotomy, it was considered that there would be no problem with anastomosis even if the resection was slightly extended. Therefore, the esophagus was additionally resected 2 cm from the cut end to obtain a sufficient and appropriate margin after dissecting the lower esophagus and lower mediastinal lymph nodes. Then, the remnant stomach was pulled out into the thoracic cavity and anastomosis of the lower esophagus and remnant stomach was performed using the double-flap technique by the hand-sewn procedure. The bottom of the exfoliated muco-submucosal layer of the remnant stomach was opened in preparation for anastomosis with the esophagus (Fig. 2A). For posterior wall anastomosis, the esophageal and muco-submucosal layers of the stomach were sutured together using knotless, barbed, absorbable sutures (V-Loc; Covidien, Mansfield, MA, USA) under retraction with a stay suture (Fig. 2B). Continuous layer-to-layer sutures were placed between the esophageal and gastric anterior walls using the V-Loc (Fig. 2C). The anastomosis was covered with a double flap, and each flap was sutured together using four or five interrupted sutures. The lower end of each flap and seromuscular layer of the remnant stomach were sutured to each other. The upper ends of the two flaps were fixed to the front wall of the esophagus (Fig. 2D). The total operative time was 613 min. The estimated blood loss was 136 mL.

**Fig. 3:**
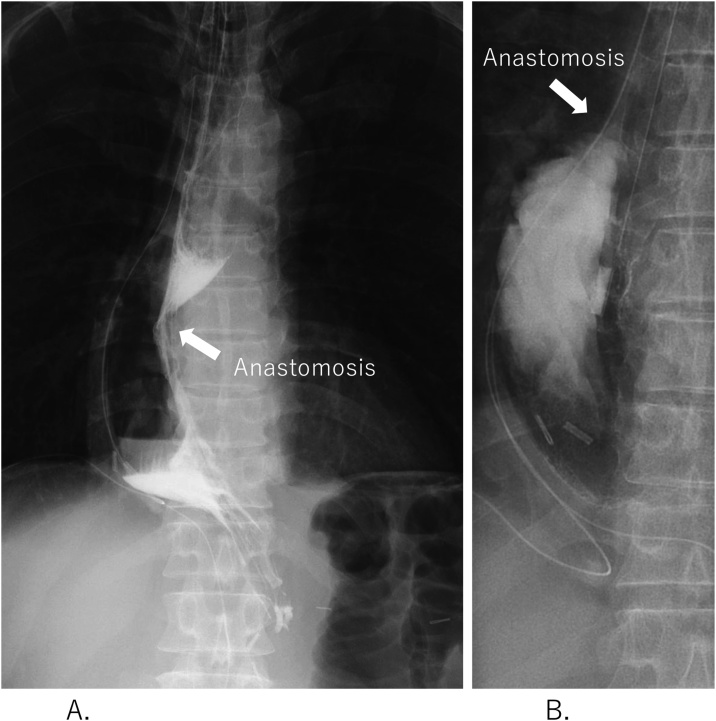
Esophagography images. **(A)** No anastomotic stenosis in the upright position. **(B)** No regurgitation of the contrast agent in the Trendelenburg position.

Oral ingestion was started on postoperative day 6. Esophagography showed no anastomotic stenosis and no regurgitation of contrast agent into the esophagus, even when the patient was placed in the Trendelenburg position (Fig. 3A, B). The patient had no complications and was discharged on postoperative day 17. We followed-up every 3 months; at 21 months postoperatively, he had not experienced heartburn, even without medication. Upper gastrointestinal endoscopy was performed every 6 months, but there were no reflux findings. Oral intake was the same as before surgery, and no weight loss was observed.

## 3 Discussion

We performed laparoscopic proximal gastrectomy, lower esophagectomy, and thoracoscopic esophagogastrostomy using the anti-reflux double-flap technique in the prone position. To the best of our knowledge, there have been few reports on this procedure. Although, there is a report that the double-flap method was performed for intrathoracic operation for thoracic esophageal cancer; however, this is the first report that thoracoscopic esophagogastrostomy was performed using the double-flap technique for EGJ cancer [5].

An optimal surgical treatment strategy for patients with EGJ cancer has not been established yet, and surgery is associated with several problems, especially for Siewert type II tumors [6]. In patients who require lower esophagectomy for EGJ cancer, we frequently have difficulty obtaining an adequate surgical margin from the abdominal cavity because of invasion into the esophagus. A safe field of view from the abdominal cavity may not be able to be secured for anastomosis depending on the length of the remaining esophagus. Therefore, intrathoracic reconstruction is frequently necessary for EGJ cancer. Additionally, severe reflux of stomach or bile acid is a common postoperative complication [7,8].

The double-flap technique has been increasingly used in the reconstruction method after proximal gastrectomy of early-stage upper gastric cancer because it has the advantage of reflux prevention and good postoperative quality of life [2,3]. The laparoscopic intrathoracic double-ﬂap technique through the transhiatal approach, which must be used to cut the left diaphragmatic crus widely, can be used to pull the right diaphragmatic crus rightward and retract the heart upward through an additional thoracic trocar to secure good vision for anastomosis. This procedure is recommended for patients with EGJ cancer with esophageal invasion of approximately 1 cm [9].

When the present procedure and conventional transhiatal approach from the abdomen are compared with regard to the treatment of EGJ cancer, the present procedure has one disadvantage: the need for repositioning during the operation. However, the merits of our technique include obtaining of a good visual field in the lower mediastinum without a wide cut of the left diaphragmatic crus, rightward pull of the right diaphragmatic crus, and upward retraction of the heart. This renders simpler procedures with guaranteed adequate surgical margins for tumors at a higher position of the lower esophagus, dissection of the lower mediastinal lymph node, and hand-sewn anastomosis in the thoracic cavity.

Although one study has reported using the intrathoracic double-flap technique for anastomosis through the open thoracoabdominal approach for EGJ cancer [10], the use of this method with the complete thoracoscopic approach has never been reported. The advantage of the laparoscopic and thoracoscopic procedure is minimal invasiveness compared to that of the open thoracoabdominal approach. Furthermore, the thoracoscopic operation in the prone position enables a good magnified field of view for thoracic procedures.

## 4 Conclusions

In summary, the double-flap technique for laparoscopic surgery and thoracoscopic esophagogastrostomy is a very useful technique due to minimal invasiveness, ease of the thoracic procedure, and prevention of reflux in patients with EGJ cancer. It is necessary to additionally evaluate the usefulness of this technique by its application in clinical and surgical settings and assessing the postoperative quality of life of patients.

## Sources of funding

The authors declare that this study received no external funding.

## Ethical approval

As a case report without Protected Health Information, no ethics approval was required for this study.

## Consent

Written informed consent was obtained from the patient for the publication of this case report and any accompanying images. A copy of the written consent is available for review by the Editor-in-Chief of this journal.

## Author contribution

MO and YH drafted the manuscript. MO, YH, ME, and KT contributed to patient care. MO and YH performed the literature search. MO, YH, ME, KT, and MO participated in the critical revision of the manuscript. All the authors have read and approved the final manuscript.

## Registration of research studies

This is a case report.

## Guarantor

Yoichi Hamai.

## Provenance and peer review

Not commissioned, externally peer-reviewed.

## Availability of data and material

The datasets generated and/or analyzed during the current study are not publicly available due to patient confidentiality reasons, but are available from the corresponding author on reasonable request.

## Declaration of Competing Interest

The authors declare no conflicts of interest.
